# General Practitioners’ Views on Communication About Dietary Supplements During Periodic Health Examinations: A Cross-Sectional Survey in Germany

**DOI:** 10.1177/21501319251333388

**Published:** 2025-05-08

**Authors:** Thomas Okon, Sascha Eickmann, Sophia Wagner, Hansjörg Baurecht, Anne Herrmann

**Affiliations:** 1 University of Regensburg, Germany; 2 Bavarian Cancer Research Center (BZKF), Germany; 3 University Hospital Regensburg, Germany

**Keywords:** dietary supplements, periodic health examination, communication, primary care, survey

## Abstract

**Introduction:**

The global dietary supplements (DS) market is expanding, numerous adults regularly consume DS. Potential interactions with prescribed medications raise concerns, but communication about DS intake during medical consultations remains limited. This study explores general practitioners’ (GPs) perceptions of communication on DS during periodic health examinations (PHEs).

**Methods:**

We conducted a cross-sectional online survey among 162 German GPs between May and August 2021. The pseudonymized web-based questionnaire assessed DS-related was carried out to analyze quantitative data.

**Results:**

In total, 162 general practitioners (GPs) participated in the survey, aged 50.2 years (±11.1). While 64.8% of GPs considered DS to be an important topic, 38.8% rarely or never (<25% of conducted PHE) addressed DS during PHEs. Personal DS use (Cramers’ *V* = 0.407; *P* < .001) and considering DS an important topic (Cramers’ *V* = 0.231; *P* = .016) were associated with more frequent discussions about DS. Time constraints (24.9%), competing priorities (21.4%), and uncertainty about DS (20.5%) were identified to be the main barriers. Suggestions for improving communication included offering more reliable information and including DS in the medication plan.

**Conclusion:**

This is the first study addressing communication about DS in Germany. Despite recognizing the relevance of DS, GPs’ communication practices remain limited due to time constraints and competing priorities. Integrating DS in the PHE could identify potential interactions with medication, strengthen patient-doctor-relationship, and satisfy patients’ needs for individualized counseling. Implementation of standardized DS documentation in medication plans and the provision of evidence-based information resources may improve patient safety and fulfill GPs informational needs.

## Introduction

The global market size for dietary supplements (DS) was estimated to exceed $170 billion USD in 2023 with the rapid growth continuing.^1^ In the largest market, the United States, nearly half of adults (49%) consume DS.^2^ In Germany, more than 20% of adults are taking DS,^3^ and this figure rises to 45% among individuals aged 65 years and older.^4^

Dietary Supplements (DS) are defined according to the Directive 2002/46/EC by the European Parliament and the Council of the European Union as: “(. . .) Food supplements means foodstuffs the purpose of which is(. . .) to supplement the normal diet and which are concentrated sources of nutrients or other substances with a nutritional or physiological effect, alone or in combination (. . .).”^5^

This definition implies that DS are not regulated as drugs,^6^ meaning a prescription from a physician is not required. Previous studies have shown that the simultaneous intake of prescribed medication and DS occurs in 19.6% to 63.7% of patients.^7^ This co-occurrence presents a significant risk for dangerous drug-supplemental interactions (eg, potassium and ACE-inhibitors; which may lead to life-threatening hyperkalemia) as demonstrated by Qato et al^2,7^ and Chiba et al.^8^ Furthermore, it is estimated that DS cause 23 000 emergency department visits annually in the United States.^9^ Consequently, the US Food and Drug Administration (FDA) recommends consulting with a physician before taking DS.^10^ The German Federal Institute for Risk Assessment (BfR) is responsible for consumer health protection in Germany. The Institute rates DS unnecessary for individuals with a balanced diet, except under special circumstances, such as pregnancy.^11^

As the primary point of contact for medical concerns, GPs play a crucial role in maintaining an overview of patients’ diseases and treatment regimens, including medications.^12,13^ However, time constraints often limit the thoroughness of this task.^14,15^ As a potential solution, the German health care system offers all patients aged 35 years and older with statutory health insurance a voluntary health check every 3 years, which should include an assessment of risk behavior and diet. Patients between the ages of 18 and 34 years are offered a 1-time PHE.^16^ Private health insurance plans often provide similar approaches.

It is well documented that patients often do not disclose their use of DS to their GPs as comprehensively as needed.^17-19^ Thus, effective communication between patients and GPs is vital to address key issues surrounding supplement consumption.^18,20,21^

Given that DS are classified as foodstuffs, a PHE represents a suitable opportunity for discussion of DS between patients and their GPs. However, quantitative data, particularly from Germany, regarding communication practices concerning supplements are lacking.^22^ Therefore, the aim of the study is to analyze the communication behaviors of physicians and their perspectives of DS during PHE.

## Methods

### Study Design and Questionnaire Development

This cross-sectional study utilized an online questionnaire focused on DS. The addressed topic was the second part of a survey about attitude,^23^ knowledge, and communication about DS. The Checklist for Reporting Of Survey Studies (CROSS) was employed to ensure comprehensive reporting of all relevant information.^24^ Following an exploratory design approach, we developed a novel questionnaire through a systematic 3-stage process. First, we conducted a comprehensive literature review in PubMed to identify relevant domains and items. Second, the questionnaire underwent multiple iterative refinement rounds with an interdisciplinary expert panel (n = 8) comprising health services researchers, clinical scientists, and practicing physicians to establish face validity. Finally, we conducted cognitive debriefing interviews with 6 physicians during a pilot phase to optimize item comprehension and response formats. Attention was given to unambiguity and avoiding misunderstanding. Adjustments were made as necessary to improve the language and clarity. According to the pilot test, the abbreviations were clarified and the language used was clear and neutral. Furthermore, the Flesch-Kincaid score was used to assess readability. A score of 10 was rated as acceptable. As a result of the pilot test, it was assumed that the participants would be able to complete the questionnaire within 10 min.

The communication part of the survey “Communication about DS during PHE” comprised 19 elements, including both multiple choice questions and single choice questions. Some questions explicitly allowed multiple responses when applicable. Communication behavior was defined in 2 categories: the general addressing of a topic during a PHE (named “topic of the consultation”) and the direct approach by the GP ( named “Direct addressing of DS use”).

Open-ended response options were included where appropriate to allow room for exploratory responses and included opportunities for free-text responses. Participants did not receive financial reimbursement. Sociodemographic data, including practice location, experience, and gender, were collected at the end of the survey. The questionnaire was designed in German. The survey was distributed during the COVID-19 pandemic as a web-based tool (REDCap^®^) and administered between May and August 2021.^23^ The trial was approved by the local ethics committee (Nr. 21-2310-101) and adhered to the principles outlined in the Declaration of Helsinki and Good Clinical Practice.

### Eligibility Criteria

Eligible participants were residents in family medicine training programs and family physicians who trained or worked, respectively, in family medicine clinics or practices and performed PHEs in Germany. In this study, the term “GPs” referred to these participants. Participants were required to have proficiency in reading and understanding German.

### Data Collection

The questionnaire was distributed among a list of GP teaching practices at different German universities and through personal networks. Prior to survey completion, digital consent was obtained, and survey data were pseudonymized in accordance with the General European Data Protection Regulation (GDPR).

### Statistical Analysis

Statistical analysis was conducted using IBM^®^ SPSS Statistics (Version 29.0.0.0, Armonk, New York). Continuous data were presented as the mean with standard deviation or as the median with quartiles, depending on their distribution, while categorical data were reported as frequencies and percentages. In cases with missing responses, percentages were calculated based on the total number of valid responses. Missing data were removed.

The strength of the correlations was calculated by Cramer’s *V* and categorized as weak (≤0.2). moderate (>0.2-0.4), relatively strong (>0.4-0.6), strong (>0.6-0.8), and very strong (>0.8).^25^ The significance of the correlations was assessed using the chi-squared test and a *P*-value of <.05 was considered statistically significant. If the chi-squared test was invalid due to at least 1 cell having an expected frequency of <5, Fisher’s exact test was used.^25^

## Results

### Study Population

A total of 162 GPs completed the survey. Since the recruitment process relied on personal networks, the sample consisted of a convenient group of GPs and therefore, a response rate be presented.

Of the participants, 43.5% were female with a mean age of 50.2 years (SD = 11.1). While 22% of the GPs had up to 10 years of experience as a doctor, 26.5% reported more than 3 decades of professional experience. Most of the GPs were located in southern regions of Germany, with more than 60% practicing in rural areas (defined as cities <20 000 inhabitants). Nearly half of the GPs (41%) reported using DS themselves. Almost two-third of the GPs (64.8%) considered DS as an important topic. The sociodemographic characteristics of the participants are outlined in Table 1.

**Table 1.** Sociodemographic Data of Participating Physicians.

	n	Mean (SD)
Age (mean)	137	50.24 (11.13)
	n	In %
Gender	147	
Female	64	43.5
Male	81	55.1
Diverse	2	1.4
Specialist	146	
Yes	124	
No	22	
Practical experience (in years)	140	
Up to 10	31	22.1
11-20	31	22.1
21-30	41	29.3
More than 30	37	26.5
Physicians’ office location (as per inhabitants)	146	
Large city (>100 000)	21	15.2
Small town (>20 000)	34	24.6
Rural area (<20 000)	83	60.1
Self-reported use of dietary supplements	151	
Yes	62	41.1
No	89	58.9

### Communication About DS During PHE

Each participant reported conducting a median of 150 (interquartile range = 100-300) PHE per year. Regarding the time spent per PHE, 60.2% of the physicians reported spending 15 to 20 min per patient and PHE, while 8.4% spent 10 min or less, and one-third of the physician (32.2%) spent more than 25 min per patient. A total of 47.1% of physicians reported spending 50% to 75% of their time of medical history and anamnesis. A summary of the overall data is presented in Table 2.

**Table 2.** Summary of Overall Periodic Health Examination Data.

Number of check-ups per year (n = 133; median; quartils)	150 (100;300)
Specified duration of check ups (n = 142)	%
Less than 10 min	2.8
10 min	5.6
15 min	29.6
20 min	29.6
More than 25 min	32.4
Percentage of medical history of PHE time (n = 140)	%
100%-90%	1.4
90%-75%	12.9
75%-50%	47.1
50%-25%	32.9
25%-0%	5.7

**Figure 1.**
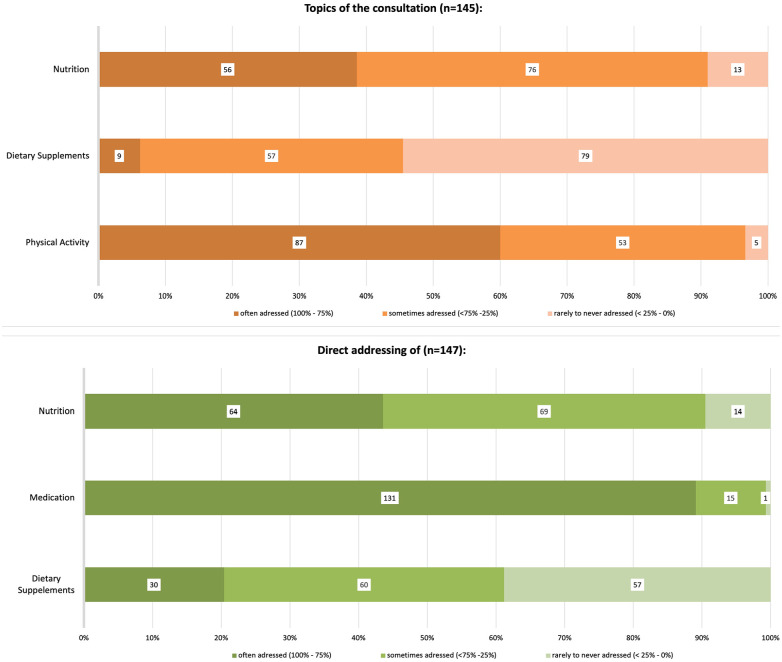
Percentage of topics discussed during a periodic health examination (above) and the direct addressing of issues by physicians (below).

Physicians were asked how often they discussed physical activity, nutrition, and DS during PHE. Additionally, the survey explored how often they actively inquired about DS, nutrition, and medication. Responses were grouped into 3 categories: addressing the issue often (in 75%-100% of the PHE), sometimes (<75%-25%), or rarely to never (<25%-0%), see Figure 1.

The results revealed that 43.5% of the physicians often discussed nutrition, while 89% of the physicians directly addressed medications during PHE in more than 75% of their PHEs. However, 38.8% of the physicians stated that they rarely or never addressed DS directly, and nearly 50% of the sample reported that the topic did not arise spontaneously during PHE.

**Figure 2.**
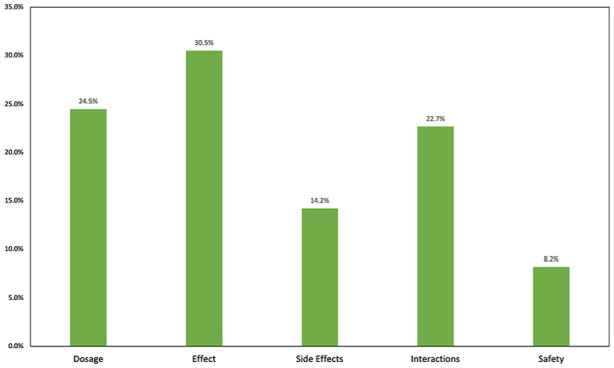
Physicians reported patient inquiries about DS.

When DS were discussed during PHEs, GPs indicated that most of the questions asked by patients were related to therapeutic effectiveness (30.5%), dosage (24.5%), and interactions with other medications (22.7%; Figure 2). When asked about the assumed reasons for their patients taking DS, the participants mentioned “support of the immune system” (18.9%), “improvement of well-being” (17.7%),“maintenance of health” (16.3%), and “enhancement of personal performance” (6.5%). Only 7.6% of GPs assumed that patients used DS for therapeutic purposes only.

If DS were discussed, 97% of the physicians stated that women are more likely to disclose their supplement use, Additionally, 86.1% of the physicians observed that physically more active patients were more likely to discuss DS use, while 89.6% noted the same for individuals with higher education levels compared to inactive individuals, men, or those with lower education.

### Correlation Analyses

**Figure 3.**
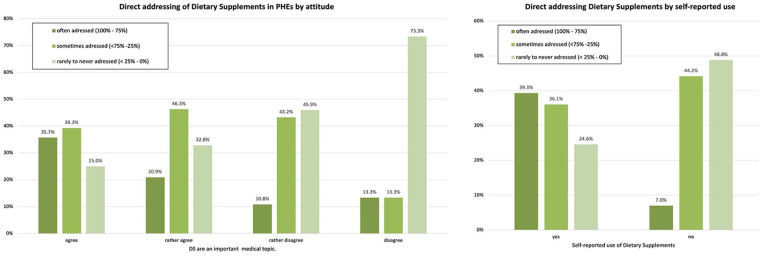
Associations of addressing DS with: (a) attitude toward DS and (b) self-reported use.

Correlation analyses were performed to examine associations between communication behavior and potential influencing factors. Sociodemographic characteristics (practice location, professional experience, and gender) showed no significant influence on the frequency of communication about dietary supplements (DS) during a PHE. Similarly, there was no significant association observed between the reported duration of a check-up and discussions about DS (Table 3). However, physicians who considered DS important^23^ were more likely to directly address them with their patients (Cramer’s *V* = 0.231; *P* < .016). Additionally, personal use of DS^23^ was significantly correlated with actively addressing the topic during a PHE (Cramer’s *V* = 0.407; *P* < .001; Figure 3).

**Table 3.** Correlation Analysis.

Potential influencing factor^a^	Correlation coefficient (“direct addressing of DS”)	*P*-value
Sociodemographic factors		
Practical experience	0.200	.169
Physicians’ office location	0.137	.272
Gender	0.153	.074
PHE-related factors		
Duration of PHE	0.143	.731
Percentage of medical history of PHE time	0.203	.183

a The categorizations were conducted based on the classifications outlined in Tables 1 and 2.

### Challenges Discussing DS Intake During PHEs

Physicians were asked why patients might avoid discussing DS. Most GPs believed that patients were concerned about their treating GP reacting negatively (17.7%) or stated that the topic of DS is not addressed because GPs do not initiate direct discussions (17.4%). Additionally, 10.2% of the respondents anticipated that patients might believe GPs were generally unwilling to engage in discussions about DS. Only 3.1% of physicians believed that patients refrain from disclosure because they already feel sufficiently informed.

**Figure 4.**
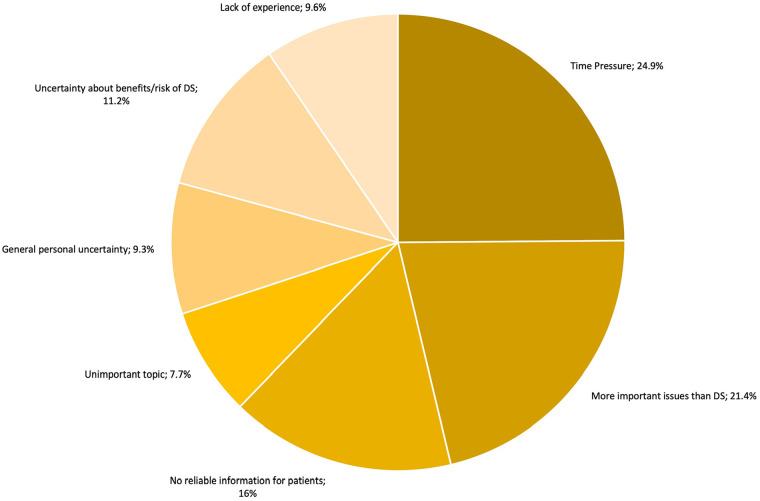
Physicians’ perspective of difficulties of addressing DS.

GPs were also asked about their own challenges they faced in addressing DS. Time constraints were cited by 24.9% of respondents, while 21.4% believed that other, more pressing health topics took priority over DS discussions. However, only 7.7% considered DS an unimportant topic. Additionally, 9.6% reported having limited experience with DS, and 20.5% expressed uncertainty, particularly regarding the risk-benefit ratio of supplements (Figure 4).

The survey included an open-text question soliciting suggestions to enhance communication regarding DS. Some respondents (n = 17) proposed providing more credible information, such as guidelines, and setting up a reliable, pharmaceutical-industry-independent platform for information. Furthermore, 2 physicians suggested incorporating DS into standardized medication plans of patients.

## Discussion

To the best of our knowledge, this is the first study to examine the communication practices and perceptions of GPs regarding DS during PHEs in Germany. The results indicate that: (I) only a small proportion of the respondents surveyed regularly address DS during PHEs, (II) considering DS an important medical topic and personal use of DS are positively correlated with discussing DS directly, and (III) the main reasons for not addressing DS include GPs not actively inquiring about DS use, time constraints and more pressing health topics and the belief that patients anticipate a negative reaction from their GP.

### PHE Communication Framework

A prevention-oriented consultation, including an anamnesis of dietary behavior is an integral part of PHEs in Germany.^16^ In terms of topics, a previous study found that over 80% of GPs considered a PHE valuable for evaluating and identifying individual risk factors. Additionally, more than 70% indicated that conducting a PHE strengthens the patient-physician relationship.^26^ In a previous trial, Approximately half of all patients in Germany with statutory health insurance participated in a PHE.^27^ Examining sociodemographic characteristics reveals a notable overlap between patients who use DSs and those who participate in PHEs, in particular those with higher socio-economic status, greater educational attainment, and higher levels of physical activity.^27-29^ This is consistent with the sociodemographic profile of supplement-taking patients reported by physicians in this study. Therefore, addressing DS by discussing supplement use directly during PHEs could be an effective way to reach the relevant patient population. Unfortunately, recent data on the content of PHEs in Germany are scarce, particularly from the perspective of GPs. Thus, our results (Figure 1) provide valuable insights into PHEs, especially in the context of the anamnesis process corroborating previous findings.^26,27,30^

The GPs primarily reported on patients’ questions regarding dosages, side effects, safety, and interactions of DS with other medications (Figure 2). From the patients’ perspective, according to previous surveys, 41% of individuals using vitamins as supplements perceived the associated health risk with consuming concentrated vitamins as (very) high or medium. Surprisingly, only 27% of this group (11.1% of vitamin supplement users) considered themselves (very) well informed about the associated health risks.^31^ This finding aligns with the perceptions of the physicians surveyed in our study, where only 3.1% of GPs believed that patients refrain from asking about DS because they feel sufficiently informed. Considering existing studies and our findings, it can be inferred that GPs are indeed aware of patients’ needs and concerns but still fail to address them adequately.

### Addressing Barriers of Communication About DS During PHE

The respondents mentioned that a lack of direct addressing is one of the reasons for non-disclosure of DS. As theoretically plausible and demonstrated by Guzman et al,^30^ directly inquiring of DS intake promotes disclosure of DS. Our results support this finding, as nearly every fifth physician mentioned a lack of direct inquiry as a reason for nondisclosure. The primary reasons for not addressing DS among physicians include time constraints, competing priorities combined with a lack of awareness regarding DS and uncertainty about the topic.

The presence of time pressure is also consistent with prior studies.^14^ In this study, most physicians spent about 15 to 20 min on a PHE, aligning with older data from Von Dem Knesebeck et al^14^ (mean 19.7 min per PHE). Thus, it would be expected that if time constraints, one of the main reasons for not addressing DS, would correlate with the duration of the PHE. Surprisingly, the duration of a PHE was not associated with directly discussing DS in our study. This may indicate that individual attitude toward DS act as an important modifier, which shows a significant moderate correlation, and thus may play a more crucial role than time constraints alone. Additionally, personal use of DS was positively correlated with actively addressing DS during PHE. A similar pattern has been observed in exercise counseling, where physicians’ own habits influence their counseling behavior.^32^

A further reason for not addressing DS is that other, more pressing health topics are given higher priority. Therefore, the potential benefits of discussing DS should be highlighted to justify its prioritization.

First, drug-supplement interactions are of interest to patients, as reflected by their assumed inquiries (Figure 2). Thus, addressing DS could be crucial to detect possible side effects and harmful interactions.^2,7,8,33^

Second, discussing DS could offer patients a more personalized health examination, a growing demand among patients,^34^ and potentially further strengthen the physician-patient relationship.^26,35-37^ Effective physician-patient communication has been shown to improve health outcomes, including blood pressure control, pain reduction, decreased psychological distress, and improved treatment adherence.^38,39^

Third, Dickinson et al^40^ reported that 1 in 4 supplement users take DS to reduce the risk of serious illness, 42% of them to improve their diet (“filling nutrient gaps”), and 58% take DS for “overall health and wellness.” Bailey et al^41^ and Iłowiecka et al,^42^ (conducted in Poland, UK, and Germany) described similar motives in their recent work, although the distribution differs slightly. When examining the reasons patients take supplements, there is notable alignment between physicians’ assumptions in this trial and the reasons patients themselves report, consistent with previous studies.^8,19,32,41,43^ Therefore, a brief discussion about DS, including an exploration of the reasons for their use, such as maintaining health and/or improving nutrition,^40,41,43^ could effectively raise awareness of unhealthy lifestyle habits, which patients may attempt to address through DS.

### Perspective

However, DS are likely to become more relevant in the future, particularly among younger adults, driven by trends on social media and the increasing focus on self-optimization.^44^ Reliable information about DS, as highlighted by the physicians in the survey, could enable GPs to address this topic more effectively during PHEs, helping to bridge the current communication gap.

Incorporating DS more prominently into the standardized medication plan, as suggested by some GPs, could help increase awareness of DS,^19^ may be an easy-to-implement measure to increase visibility,^45^ and could also save time and overcome time constraints by simplifying documentation.

### Limitations

The sampling method used in our study, which focused exclusively on specific federal states in Germany, limits the generalizability of the results. Despite the relatively high number of physicians surveyed, there remains the potential for sampling bias. Additionally, there is potential for self-report bias and recall-bias regarding the number of check-ups conducted by the physicians, as well as social desirability bias concerning the reported importance of communication and time spent discussing topics with patients. Another limitation is missing data, which were excluded and thus consequently reduced the number of responses. The sampling was conducted during the early stages of the COVID-19 pandemic, a period when supplements may have played an even more prominent role.^46^ Furthermore, discussions about lifestyle modification, including nutrition and physical activity, may not only occur during PHEs but also in other types of consultations. Despite these limitations, this study provides valuable insights into communication about DS and lays the groundwork for strategies to improve patient-physician-dialogue regarding DS in Germany.

## Conclusion

There is a notable lack of data on communication regarding DS in Germany. This explorative study provides valuable insights into GPs’ perceptions of discussing DS intake during PHE. Our findings emphasize the need for more proactive engagement by physicians in addressing supplement use with their patients. Self-intake of DS and GPs’ attitudes toward DS were found to be important factors influencing their communication behavior. Addressing this topic could reduce nondisclosure, facilitate more personalized healthcare, identify DS-medication interactions, and potentially strengthen the physician-patient relationship. The study highlights the potential benefits of incorporating DS discussions into PHEs. Future research should explore patients’ perceptions of DS communication, assessing physicians’ knowledge about DS, and identifying strategies to overcome communication barriers. Providing clear guidance for GPs on how to effectively address DS during PHEs could enhance the quality of care and foster better patient outcomes.
